# CDK6 is upregulated and may be a potential therapeutic target in enzalutamide-resistant castration-resistant prostate cancer

**DOI:** 10.1186/s40001-022-00730-y

**Published:** 2022-07-02

**Authors:** Xi Chen, Yechen Wu, Xinan Wang, Chengdang Xu, Licheng Wang, Jingang Jian, Denglong Wu, Gang Wu

**Affiliations:** 1 Department of Urology, Tongji Hospital, School of Medicine，Tongji University, 389 Xincun Road, Shanghai, China; 2 Department of Urology, Baoshan Branch, Shuguang Hospital Affiliated to Shanghai University of Traditional Chinese Medicine, Shanghai, China; 3 Suzhou Medical School of Soochow University, Jiangsu, China

**Keywords:** Differentially expressed genes, Castration-resistant prostate cancer, Enzalutamide, Hub gene, Cyclin-dependent kinase 6, Therapeutic target

## Abstract

**Background:**

Androgen deprivation therapy (ADT) is still the first-line treatment of prostate cancer (PCa). However, after a certain period of therapy, primary PCa inevitably progresses into castration-resistant PCa (CRPC). Enzalutamide (Enz) is an androgen receptor (AR) signal inhibitor which can delay the progression of CRPC and increase survival of patients with metastatic CRPC. However, the mechanisms involved in enzalutamide-resistant (EnzR) CRPC are still controversial. In the study, we used bioinformatic methods to find potential genes that correlated with the occurrence of EnzR CRPC.

**Methods:**

We collected RNA sequencing data of the EnzR CRPC cell line LNCaP (EnzR LNCaP) from GSE44905, GSE78201, and GSE150807. We found the hub genes from the three datasets. Then we tested the expression of the hub genes in different databases and the potential drugs that can affect the hub genes. Finally, we verified the hub gene expression and drug function.

**Results:**

From GSE44905, GSE78201 and GSE150807, we found 45 differentially expressed genes (DEGs) between LNCaP and EnzR LNCaP. Ten hub genes were found in the protein–protein interaction (PPI) network. The expression of hub gene and survival analysis were analyzed by different databases. We found that cyclin-dependent kinase 6 (*CDK6*) was highly expressed in both the EnzR LNCaP cell and PCa patients. Ten potential small molecules could suppress CDK6 expression as per “CLUE COMMAND” findings. Finally, we found the expression of CDK6 increased in both PCa patients’ samples, CRPC and EnzR PCa cell lines. Three potential CDK6 inhibitors, namely apigenin, chrysin and fisetin, can decrease cell proliferation.

**Conclusions:**

The study proved that the abnormal overexpression of CDK6 may be a reason behind EnzR CRPC occurrence and suppression CDK6 expression may help treat EnzR CRPC.

**Supplementary Information:**

The online version contains supplementary material available at 10.1186/s40001-022-00730-y.

## Background

Prostate cancer (PCa) has the highest incidence among all types of cancers in older men in America. In 2019, the estimated incidence rate of PCa in the United States was 174,650, which amounts to a 20% incidence of all new cancer diagnoses in men. Additionally, the mortality rate associated with PCa has risen in recent years. In America, lung, prostate and colorectal cancers account for the greatest number of deaths in men [1]. Recently, the incidence and death rate of PCa have also increased in China. According to the National Cancer Center of China, 72,000 new patients were diagnosed with PCa in China in 2015, with an estimated incidence of 10.23/100 000 in men [2]. Androgen deprivation therapy (ADT) remains the first-line therapy for treating locally advanced and even metastatic PCa [3]. As an important method for treating PCa, ADT can effectively release clinical symptoms of patients and prolong survival time [4]. However, after a certain period of treatment, nearly all patients will inevitably develop castration-resistant PCa (CRPC) [5]. When patients relapse into the CRPC stage, the median survival rate of patients is less than 20 months [6]. Therefore, the treatment of CRPC is challenging.

Enzalutamide (Enz), a second-generation androgen receptor (AR) inhibitor, has been approved by the Food and Drug Administration (FDA) to treat CRPC [7]. It inhibits the nuclear translocation of activated AR and prevents its interaction with androgen response elements (ARE) and recruitment of coactivators, thereby promoting apposes while suppressing the proliferation of CRPC cells [8, 9]. Compared to placebo and bicalutamide Enz can effectively prolong the survival time of patients with CRPC [3, 10]. However, after a period of therapy, patients become resistant to Enz. No effective treatment regimens for Enz-resistant (EnzR) CRPC exist.

EnzR CRPC is associated with abnormal gene expression which plays an important role in the occurrence of EnzR CRPC. Bioinformatic analysis has been widely used for identifying potential gene mutations in this process. In this study, we found differentially expressed genes (DEGs) in the EnzR PCa cell line LNCaP (EnzR LNCaP) using the GEO database. Then, we analyzed the significant functional modules and pathways enriched in the DEGs. The hub genes were involved in a protein–protein interaction (PPI) network. The expression of the hub gene’ was analyzed by using The Cancer Genome Atlas (TCGA) and Chinese Prostate Cancer Genome and Epigenome Atlas (CPGEA) databases. Cyclin-dependent kinase 6 (*CDK6*), a hub gene, was upregulated in both EnzR LNCaP and PCa samples. Moreover, CDK6 was upregulated in clinical PCa samples and EnzR PCa cell lines. Furthermore, three CDK6 inhibitors, namely apigenin, chrysin, and fisetin, can suppress CDK6 expression and cell proliferation.

## Methods

### Data source

The GEO database (http://www.ncbi.nlm.nih.gov/geo/) at the National Center for Biotechnology Information (NCBI) is a public database that serves as a genomics data repository of gene expression, ChIP, and microarray data [11]. Because the collection of clinical samples was difficult, the RNA sequencing (RNA-seq) data of EnzR LNCaP were downloaded. The study criteria were as follows: (1) there is a noticeable distinction between PCa cells that have been treated with Enz and those that have not; (2) each group had at least three samples; (3) the sequence information was comprehensive and could be obtained from the database. Three gene expression datasets, GSE44905, GSE78201, and GSE150807, met our criteria and were downloaded from the GEO database. These datasets were included in the high-throughput sequencing of EnzR LNCaP cells and normal control LNCaP cells. GSE44905 included three control LNCaP samples and six EnzR LNCaP samples. GSE78201 included four untreated LNCaP samples and four EnzR LNCaP samples. GSE150807 included three parental LNCaP samples and three EnzR LNCaP samples. In addition, two datasets, GSE151083 and GSE136130, which have the RNA-seq data of EnzR C4-2 samples were downloaded.

### Identification of DEGs

The associated genes were mapped to raw microarray expression data of mRNAs obtained as Series Matrix files from the GEO database using SOFT-formatted family files from the GEO database. The primary data were normalized by R the ‘limma’ package [12]. The genes with an adjusted *P*-value < 0.05 and |log2-fold change (FC)|> 1 were considered DEGs [13]. Then, an online web tool, bioinformatic (http://www.bioinformatics.com.cn/), was used to draw a Venn map and identify the DEGs. The upregulated and downregulated DEGs were retained for further analysis.

### GO and KEGG analyses

GO terms include biological processes (BP), molecular functions (MF), and cellular components (CC). In addition, KEGG pathways are widely used to analyze biological pathways associated with DEGs. Both GO annotation and KEGG pathway enrichment analyses were performed using Metascape (http://metascape.org/). Then, scatter plots were drawn using an online web tool, bioinformatic (http://www.bioinformatics.com.cn/). Values with *P*-value < 0.05 were considered statistically significant.

### Construction of PPI network and identification of hub genes

To evaluate the potential PPIs among the identified DEGs, the STRING online database (http://string-db.org/) was used to build a PPI network. Then, Cytoscape software was used to find the correlation among DEGs. The hub gene was defined as a gene whose expression was correlated with that of other genes.

### Detection of hub gene expression

TCGA, a large tumor database, includes information of 499 PCa samples and 52 normal prostate samples [14]. GEPIA (http://www.gepia.cancer-pku.cn/) can identify the expression of hub genes in TCGA samples and samples from the Genotype-Tissue Expression (GTEx) database. We sought to confirm the expression of these genes in Asian populations because the TCGA database mostly contained information from the Western population. Therefore, CPGEA (http://www.cpgea.com/), which included the RNA sequences and clinical data of the Chinese population in the study, was chosen.

### Survival and clinical value analyses

In addition to expression data, GEPIA website can be used for survival analysis as well. Survival analysis was performed using GEPIA. The correlation between hub gene expression and overall survival (OS) and disease-free survival (DFS) was analyzed. To assess hub gene expression at different tumor stages, the clinical data of patients with PCa were downloaded from TCGA. Hub gene expression was analyzed in different tumor stages according to the Tumor-Node-Metastasis (TNM) tumor classification system of malignant tumors.

### Screening of potential small molecules

CLUE COMMAND (https://clue.io/command) has genome-wide transcriptional expression data that can help find functional connections among drugs, genes, and diseases [15]. Expression data of all potential small molecules that may affect the function of the hub genes were downloaded.

### Tissue samples

Fourteen paired PCa samples and para-cancerous samples were collected at Tongji Hospital, School of Medicine, Tongji University. The methods used for collecting samples were approved by the Ethics Committee of Tongji hospital (SBKT-2021-220). Written informed consent was obtained from all patients.

### Cell culture and drug treatment

The human normal prostate epithelial cell line RWPE-1 and PCa cells LNCaP, C4-2, and 22Rv1 were cultured in Roswell Park Memorial Institute (RPMI) 1640 (Sigma Darmstadt, Germany) with 10% fetal bovine serum (FBS) (Gibco, Thermo Fisher Scientific, Waltham, MA, USA). All cell lines were cultured in a humid environment containing 5% CO_2_ and 95% air at 37 °C. The EnzR LNCaP and C4-2 cell lines were treated with Enz at 10, 20, 30, and then 40 µM until 20 days. Then, 10 µM Enz was added to make the cells resistant to Enz. After cell culture, the cell lines were treated with apigenin, chrysin, and fisetin (SelleckChem, Houston, TX, USA). These small molecules were added to the culture medium of PCa and EnzR PCa cells according to the manufacturer's instructions.

### RNA extraction and qRT-PCR

RNA from total tissues and cells was isolated using TRIzol reagent (Sigma Darmstadt, Germany) in accordance with the manufacturer’s instructions. The mRNA was reverse transcribed to cDNA using the Advantage^®^ RT-for-PCR Kit (Takara Bio Inc., Kusatsu, Japan), according to the manufacturer’s instructions. qRT-PCR was performed using the Applied Biosystems 7500 Sequence Detection system. The volume of cDNA was detected using qRT-PCR reagents and the TB Green^®^ Premix Ex Taq™ II Kit (Takara Bio Inc.), according to the manufacturer’s instructions. β-tubulin was used as a normalizing control. The expression of RNA was calculated according to the 2^−ΔΔ*Ct*^ method. The following primers were used: cyclin-dependent kinases 6 (*CDK6*) (forward: 5′-CAAGGTCAGGTCTACTCAAAGTCTCAC-3′, reverse: 5′-CTGCCAACGATTGAATGCCAGAATG-3′) and *β-tubulin* (forward: 5′-TGGACTCTGTTCGCTCAGGT-3′, reverse: 5′-TGCCTCCTTCCGTACCACAT-3′).

### Western blot

Proteins from clinical samples and cell lines were extracted using RIPA lysis buffer. Protein samples were treated with Dual Color Protein Loading Buffer (Thermo Fisher Scientific, Waltham, MA, USA). Proteins were separated using SDS-PAGE gels (10%) and then transferred to nitrocellulose membranes (NC) (Merck KGaA, Darmstadt, Germany). The membranes were then blocked with Protein-Free Rapid Blocking Buffer (Thermo Fisher Scientific) and incubated at 4 °C overnight with primary antibodies against CDK6 (1:1000) and β-tubulin (1:1000) (Abcam, Cambridge, UK). The next day, the membranes were washed with 1 × TBST three times (10 min each). Then, the membranes were incubated at normal temperature for 1.5 h with a matched secondary antibody (HRP-labeled Goat Anti-Human IgG [H+L], Beyotime Biotechnology, Shanghai, China). Finally, the membranes were exposed to an X-ray film.

### Cell proliferation assay

Cell proliferation ability was detected using a CCK-8 kit (Dojindo, Japan). In brief, the cells in 96-well plates (3000 cells/well) were cultured in 200 µL of RPMI-1640 supplemented with 10% FBS for 0, 24, 48, or 72 h. After culture, cells were counted using the CCK-8 kit according to the manufacturer’s instructions, and absorbance at 450 nm was measured using a spectrophotometer (LD942, Beijing, China).

### Statistical analysis

All experiments were repeated at least three times. The data were represented as the mean ± standard deviation (SD). Differences between two groups were analyzed using a Student's *t*-test, and differences among three or more groups were analyzed using a one-way analysis of variance. The ChIP data from GEO, TCGA, and CPGEA databases were analyzed by R software with different packages (R Version 4.0.3). Values with *P* < 0.05 were considered statistically significant.

## Results

### Identification of DEGs

**Fig. 1:**
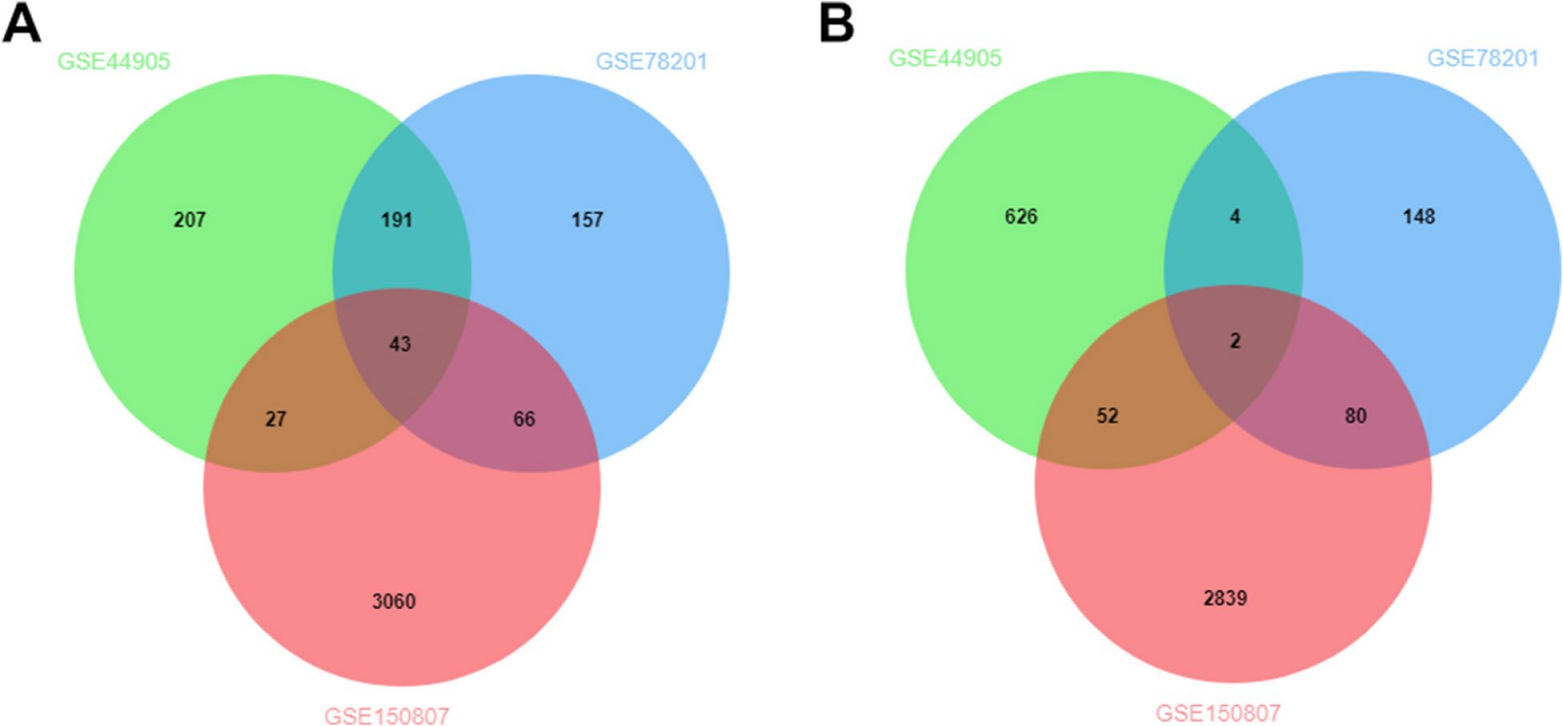
The DEGs which had differential expression between normal LNCaP cells and EnzR LNCaP cells from GSE44905, GSE78201 and GSE150807 datasets. **A** The upregulated DEGs between normal LNCaP cells and EnzR LNCaP cells from GSE44905, GSE78201 and GSE150807. **B** The downregulated DEGs between normal LNCaP cells and EnzR LNCaP cells from GSE44905, GSE78201 and GSE150807

**Fig. 2:**
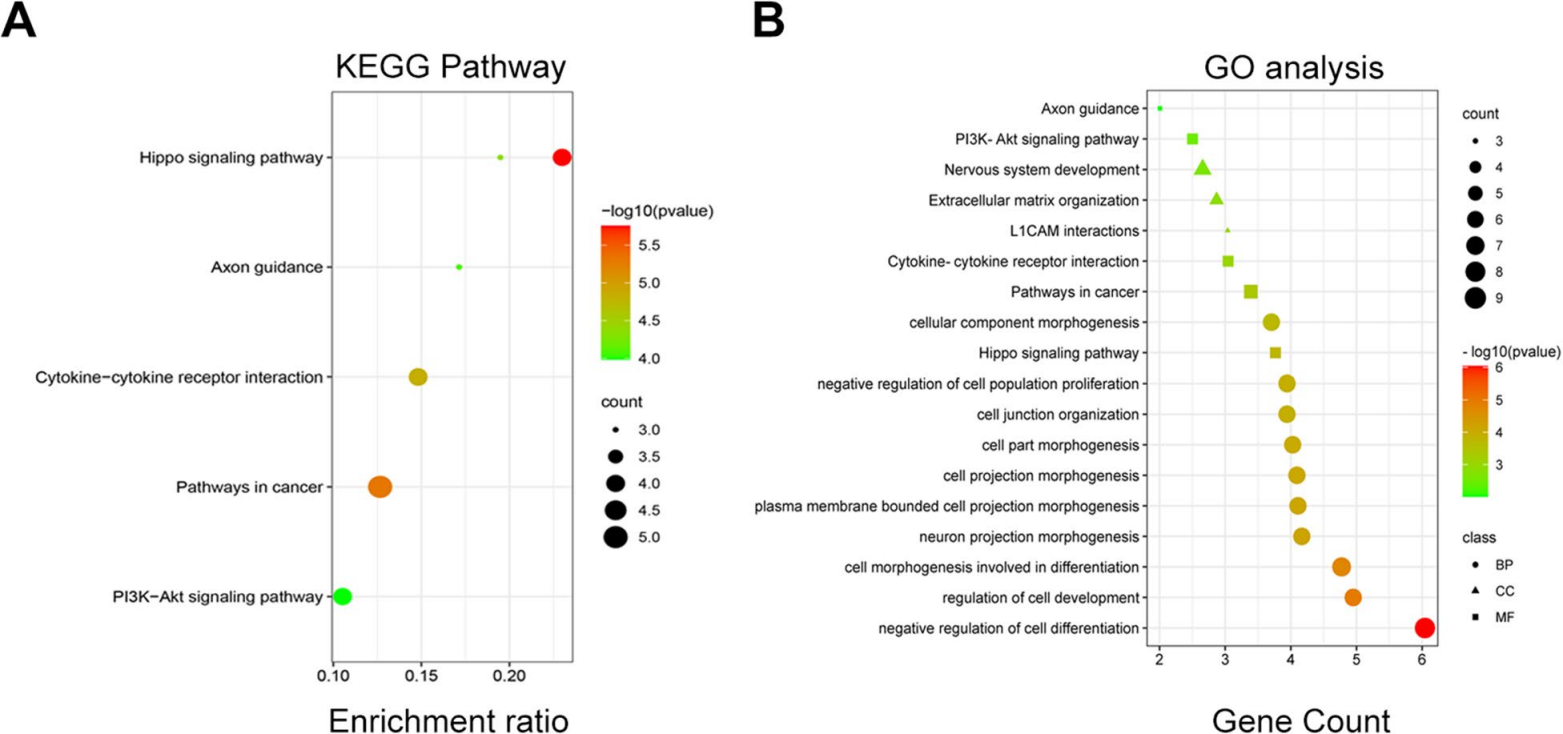
The scatter map reflects KEGG pathways and GO analysis of DEGs in enrichment analyses. **A** The KEGG pathways which the DEGs enriched was shown in scatter map. **B** The GO analysis which the DEGs enriched was shown in scatter map

We searched the GEO database to obtain EnzR PCa samples. Finally, cell samples treated by Enz were included in the study. To identify DEGs, we analyzed three gene expression datasets: GSE44905, GSE78201, and GSE150807, which overall included 10 untreated LNCaP cell samples and 13 EnzR LNCaP cell samples. In these samples, we identified 43 upregulated DEGs (Fig. 1A) and 2 downregulated DEGs (Fig. 1B). Information on these 45 DEGs is provided in Additional file 1: Table S1. Furthermore, we constructed a volcano map to reflect the DEGs among the three datasets (Additional file 2: Figure S1). A total of 45 DEGs were identified among all three datasets.

### Functional and enrichment analyses

To identify the biological functions enriched in the DEGs, we used Metascape to construct KEGG pathways and GO functional analysis. Then, we used a webtool, bioinformatic, to draw a scatter plot map. Results of GO enrichment suggested that the DEGs were primarily involved in the negative regulation of cell differentiation and cell morphogenesis. Results of KEGG pathway enrichment revealed that most of the enriched pathways were those associated with cancer development and cytokine–cytokine receptor interaction. The significant KEGG pathways that are enriched in the DEGs are shown in a scatter plot map (Fig. 2A). In addition, BP, MF, and CC pathways were also shown in the scatter plot map (Fig. 2B).

### PPI network construction and hub gene identification

**Fig. 3:**
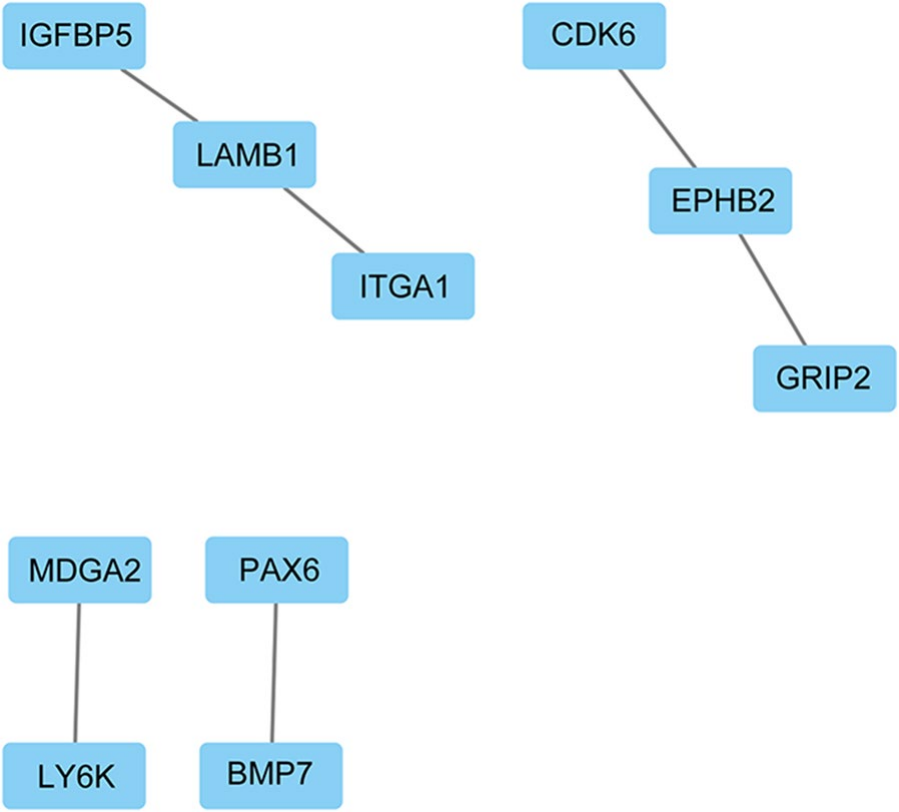
The protein–protein interaction (PPI) network of DEGs constructed by cytoscape

Next, we used Cytoscape to create a PPI network of the DEGs using the data from STRING. We created the network to find correlations among DEGs and identify hub genes (Fig. 3). We defined the hub genes as the genes correlated with other genes in terms of expression. We found that the expression of *GRIP2*, *EPHB2*, *CDK6*, *PAX6*, *BMP7*, *ITGA1*, *LAMB1*, *IGFBP5*, *LY6K,* and *MDGA2* was correlated with that of other genes. Hence, these genes were considered as hub genes.

### Hub gene function in PCa samples from public databases

**Fig. 4:**
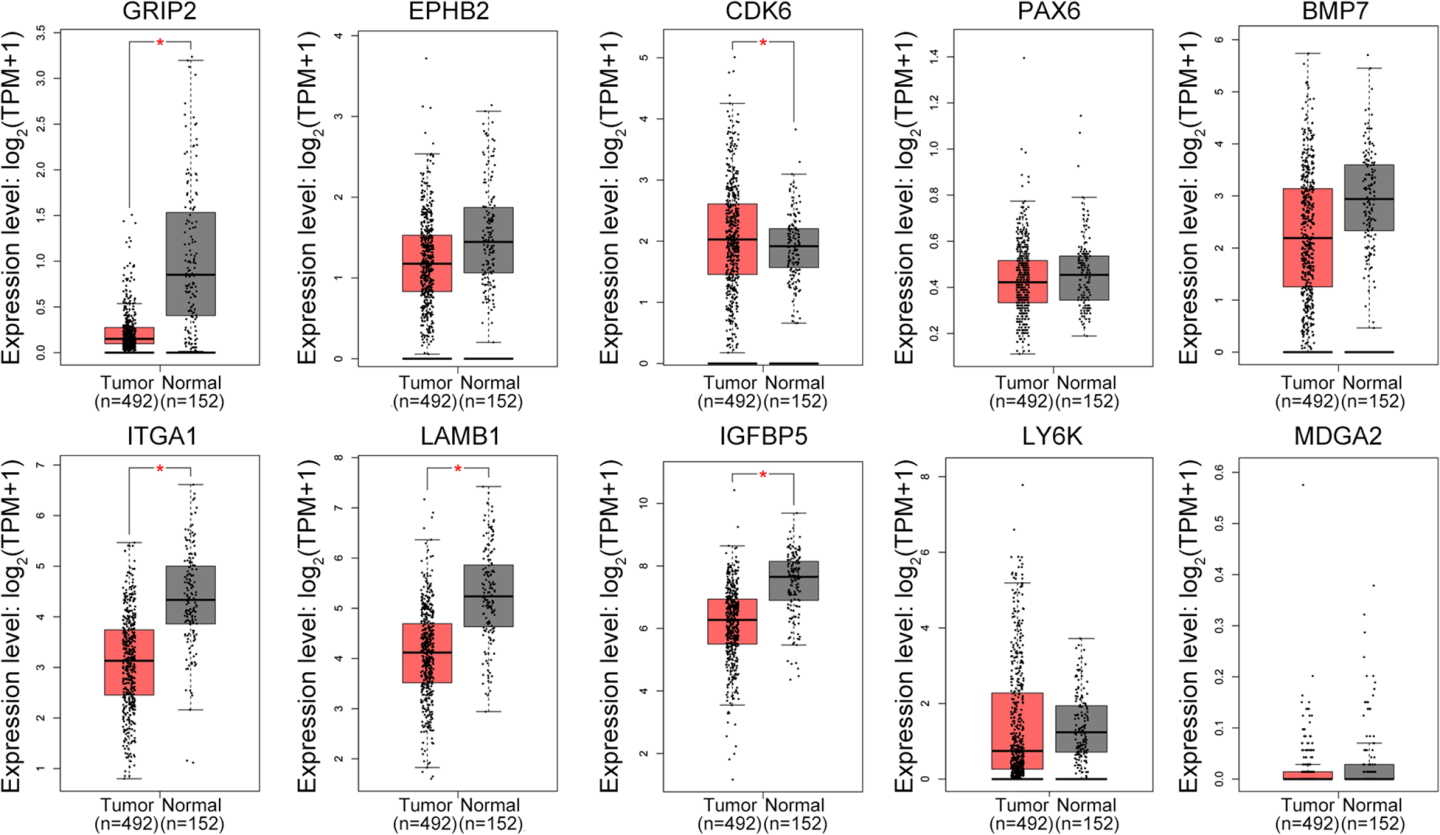
The expression of ten hub genes between normal prostate tissues and PCa samples from GEPIA online webtool (normal tissues include data from TCGA and GTEx database). *represents statistical differences. Red color represents tumor samples and gray color represents normal samples

Because the hub genes were expressed in the EnzR PCa cell line LNCaP, we tested the hub gene expression on TCGA data. We used GEPIA, which had sequencing information of all the 492 samples from patients with PCa and 152 normal controls from TCGA and GTEx databases. We found that the expression of *EPHB2*, *PAX6*, *LY6K,* and *MDGA2* did not differ, whereas that of all other genes was different in patient tissues. However, only *CDK6* was expressed highly in cancer tissues, which was in agreement with the results obtained from cell line samples (Fig. 4). The expression of other genes, which were highly expressed in the EnzR LNCaP cell line, was lower in cancer tissues than in normal tissues. In addition, we also collected the RNA-seq data from patients with PCa in the Chinese population. We found that except *LY6K* and *MDGA2,* all other hub genes were differentially expressed between PCa tissues and para-carcinoma tissues. Similar to the results from TCGA, CDK6 was highly expressed in PCa tissues (Additional file 3: Figure S2). These results indicated that CDK6 might be a potential cause of PCa. Moreover, we analyzed CDK6 expression at different tumor stages (TNM tumor stages) in patients with PCa from the TCGA database. However, we found that changes in CDK6 expression were not reflective of disease progression (Additional file 4: Figure S3). Next, we tried to find whether phenotype changes associated with CDK6 expression could affect PCa progression. We downloaded CDK6 methylation data and found that CDK6 methylation was higher in tumor tissues than in normal samples and that CDK6 methylation was correlated with the expression of *CDK6* mRNA in PCa (Additional file 5: Figure S4A, B).

### CDK6 expression in EnzR CRPC cells and its correlation with immune infiltrates

Because the LNCaP PCa cell line is derived from hormone-sensitive PCa, it cannot precisely mimic the CRPC model. Therefore, we further studied the expression of CDK6 in other PCa cell lines. As both C4-2 and 22Rv1 cell lines can grow independently without androgens, they can serve as CRPC cell lines [16, 17]. We collected RNA-seq data from two datasets, GSE151083 and GSE136130, which included the RNA-seq data from the C4-2 and EnzR C4-2 cell lines, respectively. We found that CDK6 expression was higher in the EnzR C4-2 cells than in C4-2 cells (Additional file 6: Figure S5A, B). Next, using an online webtool, TIMER (http://timer.cistrome.org/), we analyzed the correlation of CDK6 expression with immune cell infiltration. We found that CDK6 expression was associated with the infiltration of many types of immune cells in PCa (Additional file 7: Figure S6). These findings suggested that CDK6 might be a factor in the development of EnzR CRPC and that it was linked to immune infiltrates.

### Survival analysis

**Fig. 5:**
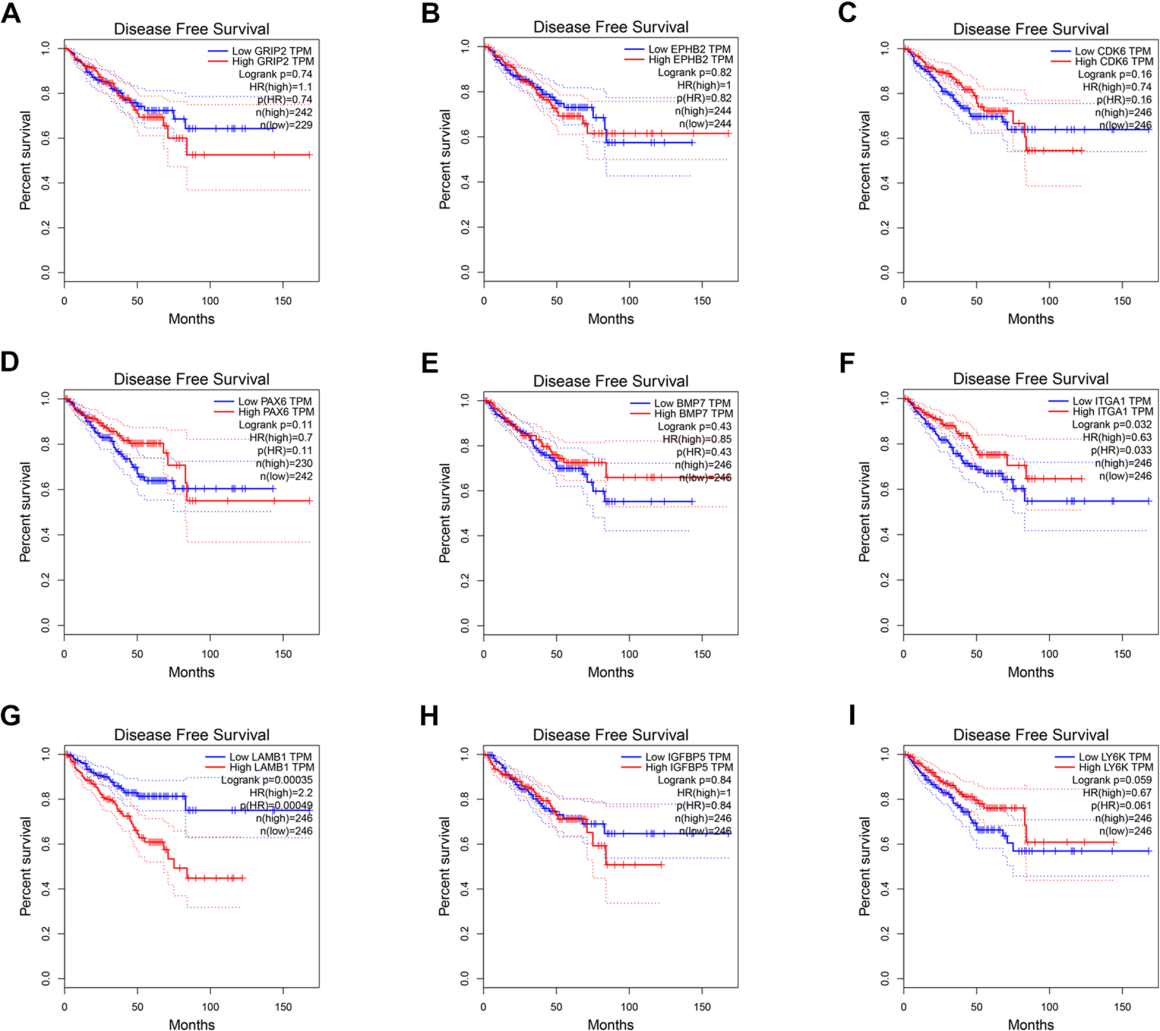
The correlation between nine hub genes’ expression and the prognosis of PCa in DFS status got from GEPIA online tool. **A** *GRIP2,* **B** *EPHB2,* **C** *CDK6,* **D** *PAX6,* **E** *BMP7,* **F** *ITGA1,* **G** LAMB1, **H** IGFBP5, **I** LY6K

We further used GEPIA to assess whether the hub genes can affect the survival status of patients with PCa. The effect of hub genes on OS and DFS was analyzed in the study. As the sample size was insufficient, we did not analyze the effect of *MDGA2*. We found that the hub genes *ITGA1* and *LAMB1* affected the DFS (Fig. 5). However, these genes could not affect the OS (Additional file 8: Figure S7). Furthermore, we discovered that CDK6 methylation did not affect OS and DFS (Additional file 5: Figure S4C, D). ITGA1 and LAMB1 appear to play a critical role in PCa incidence and development, according to the findings.

### Screening of potential small molecule drugs

Next, we attempted to identify drugs that act against EnzR CRPC by inhibiting the function of CDK6. From CLUE COMMAND, we identified 10 compounds that could inhibit the activity of CDK6 (Additional file 9: Figure S8). Three of them have been clinically used to treat breast cancer. Two compounds are currently in the preclinical phase, two in phase II, and three in phase I. The detailed information of the 10 compounds is provided in Table 1. Apigenin, chrysin, and fisetin solely affect CDK6 expression, whereas other chemicals also affect the expression of other CDK genes. Therefore, we chose these three compounds for future investigation.

**Table 1:** The detailed information of 10 CDK6 inhibitors from CLUE COMMAND

Name	Mechanism of action (MOA)	Target	Phase
Abemaciclib	CDK inhibitor	CDK4, CDK6	Launched
Alvocidib	CDK inhibitor	CDK1, CDK2, CDK4, CDK5, CDK,6 CDK7, CDK8, CDK9, EGFR, PYGM	Phase 2
AMG-925	CDK inhibitor, FLT3 inhibitor	CDK4, CDK6, FLT3	Phase 1
Apigenin	Casein kinase inhibitor, cell proliferation inhibitor	AKR1B1, AR, CDK6, CFTR, CYP19A1, CYP1B1, HSD17B1	Preclinical
AT-7519	CDK inhibitor	CDK1, CDK2, CDK4, CDK5, CDK6, CDK9	Phase 2
Chrysin	Breast cancer resistance protein inhibitor	AKR1B1, CDK6, CYP19A1, CYP1B1	Phase 1
Fisetin	Aurora kinase inhibitor	CDK6, FASN	Preclinical
Palbociclib	CDK inhibitor	CDK4, CDK6	Launched
RGB-286638	CDK inhibitor	CDK1, CDK2, CDK3, CDK4, CDK5, CDK6, CDK7, CDK9, FLT3, GSK3B, JAK2, MAP3K7, MAPK9	Phase 1
Ribociclib	CDK inhibitor	CDK4, CDK6	Launched

### Validation of CDK6 expression in clinical PCa samples and EnzR PCa cell lines

**Fig. 6:**
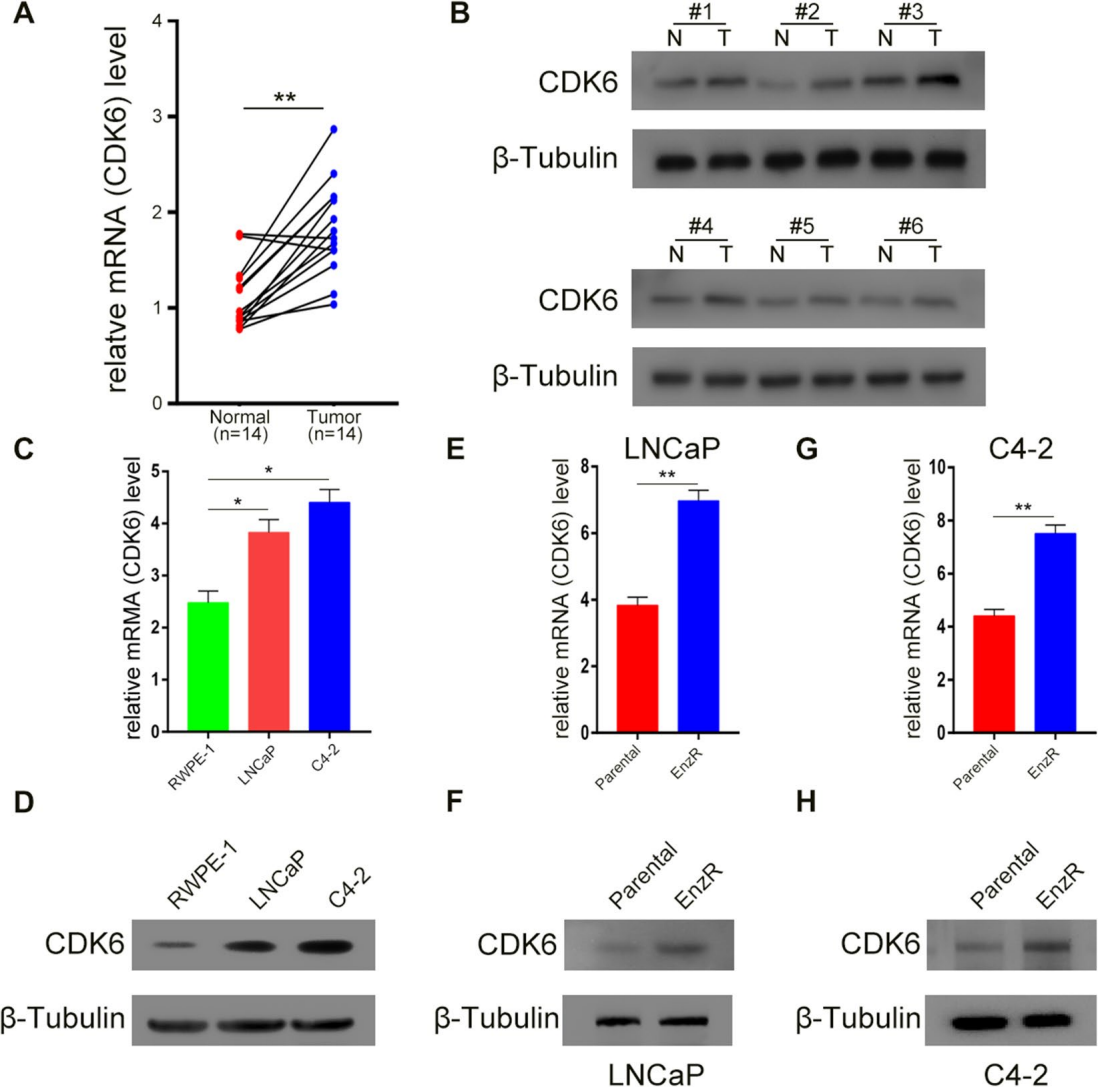
CDK6 had a higher expression in clinical PCa samples, PCa and EnzR PCa cells. **A** The mRNA expression of CDK6 in PCa tissues and para-cancerous normal tissues of 14 paired patients from Tongji Hospital. **B** The protein level of CDK6 between clinical PCa samples and para-cancerous normal tissues from 6 paired patients. **C**, **D** The mRNA and protein expression of CDK6 among normal prostate epithelial cell RWPE-1 and PCa cell lines LNCaP, and C4-2. **E**, **F** The mRNA and protein level of CDK6 between LNCaP cells not treated Enz and EnzR LNCaP cells. **G**, **H** The mRNA and protein expression of CDK6 between normal C4-2 cells and EnzR C4-2 cells. *represents *P* < 0.05, **represents *P* < 0.01. The data were shown in mean ± SD. The qRT-PCR and western blot using β-tubulin as inner control. N represents normal tissues and T represents tumor tissues. Parental means cells sensitive to Enz; EnzR means cells resistant to Enz

As CDK6 may be important in the development of PCa and EnzR CRPC, we collected PCa tumor tissues and para-cancerous tissues and EnzR PCa cell lines to compare the CDK6 expression among them. We found that the expression of CDK6 was high in PCa tissues at mRNA and protein levels (Fig. 6A, B). In addition, CDK6 expression was higher in the PCa cell lines LNCaP and C4-2 than in RWPE-1 (Fig. 6C, D). Furthermore, CDK6 expression was higher in EnzR LNCaP and C4-2 cell lines than in normal LNCaP and C4-2 cell lines (Fig. 6E, F). Due to the expression of AR splice variants, 22Rv1 cells can become resistant to Enz [18]. To see if CDK6 expression changes when Enz is introduced to the 22Rv1 cell line, we chose this cell line. We found that CDK6 was expressed higher in the 22Rv1 cell after treatment with Enz (Additional file 6: Figure S5C, D). The results suggest that CDK6 plays a critical role in the occurrence of PCa and even EnzR CRPC.

### Function of CDK6 inhibitors in PCa, CRPC, and EnzR CRPC cells

**Fig. 7:**
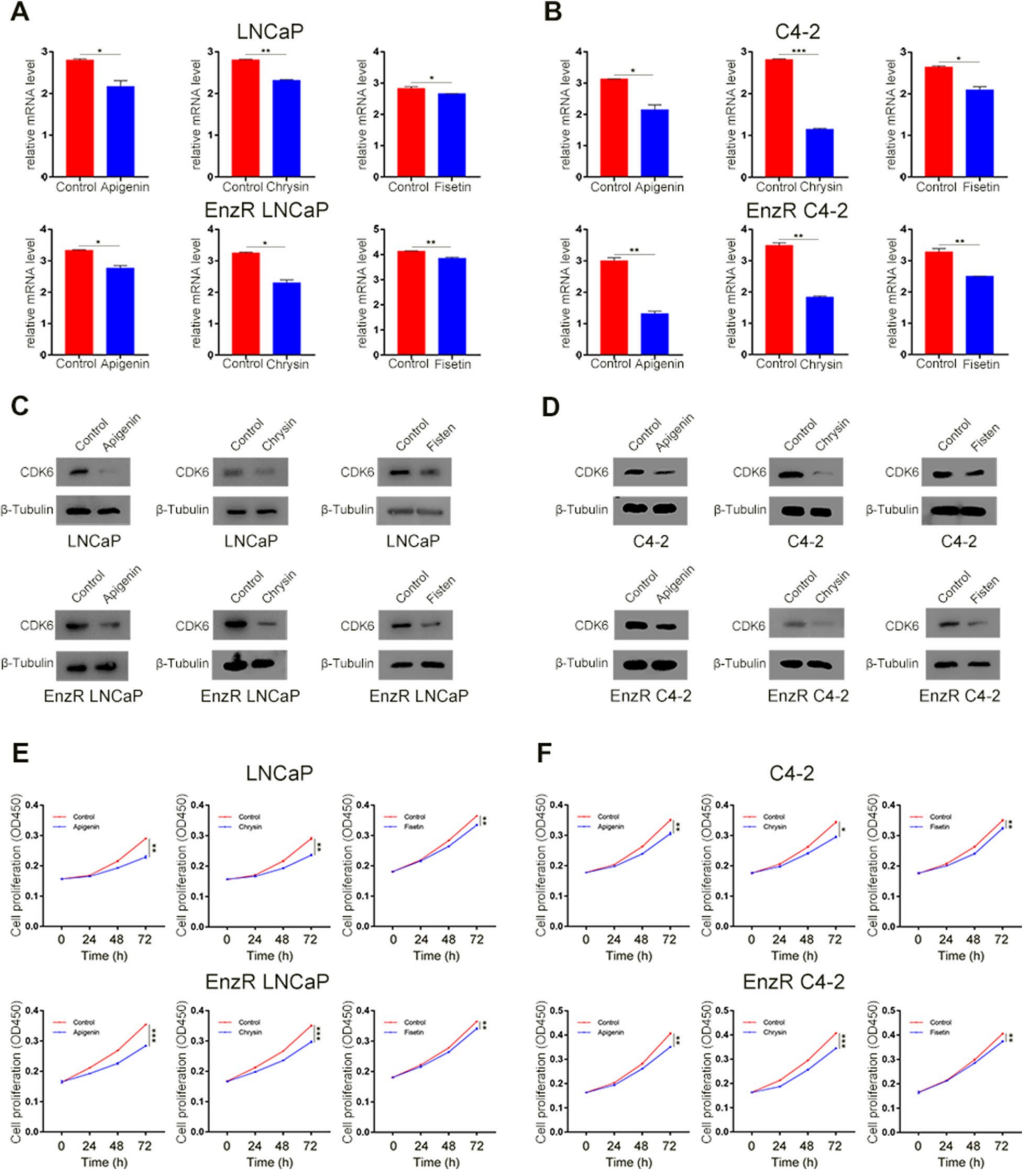
CDK6 inhibitors decreased the expression of CDK6 and suppressed cell proliferation in both PCa and EnzR PCa cells. **A** The mRNA expression of CDK6 after LNCaP cells and EnzR LNCaP cells treated by CDK6 inhibitors (apigenin, chrysin, fisetin). **B** The expression of CDK6 in mRNA level after C4-2 cells and EnzR C4-2 cells treated by CDK6 inhibitors. **C** CDK6 inhibitors can suppress CDK6 protein expression in LNCaP cells and EnzR LNCaP cells. **D** The CDK6 protein level decreased after CDK6 inhibitors treated C4-2 cells and EnzR C4-2 cells. **E** The cell proliferation was decreased after ordinary LNCaP and EnzR LNCaP treated by CDK6 inhibitors by CCK-8. **F** Normal C4-2 and EnzR C4-2 cells proliferation ability was decreased after CDK6 inhibitors used. *represents *P* < 0.05, **represents *P* < 0.01, ***represents *P* < 0.001. The data were shown in mean ± SD. The qRT-PCR and western blot using β-tubulin as inner control

To determine whether the abovementioned drugs, apigenin, chrysin, and fisetin, can treat PCa, CRPC, and even EnzR CRPC, we treated LNCaP, C4-2, EnzR LNCaP, and EnzR C4-2 cell lines with these compounds. CDK6 inhibitors significantly downregulated the expression of CDK6 at both mRNA and protein levels in PCa cell lines (Fig. 7A–D). The result suggests that these drugs can suppress CDK6 expression. In addition, when treated with these CDK6 inhibitors, the progression of PCa and EnzR PCa cells was decreased (Fig. 7E, F). As the LNCaP cell line cannot serve as a CRPC cell line, we further tested the expression of CDK6 in the 22Rv1 cell line, which is considered an EnzR CRPC cell line. We found that these CDK6 inhibitors could suppress the expression of CDK6 and cell proliferation ability in 22Rv1 cells (Additional file 10: Figure S9A–C). The results suggest that CDK6 inhibitors are protein drug candidates for the treatment of PCa, CRPC, and even EnzR CRPC in the future.

## Discussion

Millions of elderly men are diagnosed with PCa annually. In addition, PCa-related mortality has risen dramatically [1]. Similarly, the incidence of PCa has been increasing in the Chinese population, posing a serious threat to public health [2]. The current treatment methods for localized PCa include surgical prostatectomy, chemotherapy, immune therapy, and ADT [19]. To date, ADT remains the first-line therapy for treating PCa [3]. ADT effectively improves the survival of patients [20]. However, the primary PCa inevitably relapses into CRPC [5]. As a second-generation AR inhibitor, Enz can prolong the survival time of patients with CRPC by approximately 4.8 months when patients progress to the CRPC stage [21]. However, patients become resistant to Enz later. Therefore, it is important to find effective methods to treat EnzR CRPC.

Genetic changes are suggested as a key factor leading to EnzR CRPC. A study has shown that AR is expressed and is transcriptionally active in EnzR CRPC [22]. Some genes, such as kallikrein-related peptidase 3 (*KLK3*) and transmembrane protease serine 2 (*TMPRSS2*), which can activate AR show increased expression in EnzR CRPC. The occurrence of EnzR CRPC can be attributed to changes in the expression of AR target genes [23]. In patients with CRPC, AR transcription leads to the generation of splice variants. AR can perform selective shear to form hypotypes that consist of exons 1–3 with LBD deletion. The AR without LBD will fail to respond to Enz [24]. Changes in gene expression can increase the expression of AR-Vs, which mainly refers to AR-V7 in EnzR CRPC. Changes in the expression of splicing factor *hnRNPA1*, the long non-coding RNA Malat1 (lncRNA *Malat1*), arginine vasopressin receptor 1a (*AVPR1A*), and monoamine oxidase-A (*MAO-*A) can affect the expression of *AR-V7* [25–27]. Additionally, some genes that regulate Wnt signaling, such as *SOX9* and *PRKAR2B*, can also cause EnzR CRPC [28–30]. All these findings indicate that gene alteration is important in the occurrence of EnzR CRPC. Furthermore, according to a study, emerging approaches such as high-throughput next-generation sequencing can aid in the discovery of drug-resistant genes and the processes that underpin Enz resistance [31].

Bioinformatics analysis can be used to comprehensively analyze the results of multiple high-throughput sequencing data and more accurately analyze differential gene expression in the context of diseases [32]. In this study, we used bioinformatics analysis to identify the key genes and their functions in EnzR CRPC, obtaining a total of 45 DEGs and 10 hub genes. We found that a hub gene, *CDK6*, was highly expressed in EnzR PCa cell lines and patients with PCa. Finally, we showed that three potential molecules, namely apigenin, chrysin, and fisetin, could decrease the expression of CDK6 and suppress cell proliferation.

CDK6 is a CDK comprising 13 different serine/threonine kinases that become periodically activated when bound to cyclins, their respective regulatory subunits. CDKs affect various important cellular processes, including cell cycle progression and transcription. Abnormal kinase activation leads to disordered cell regulation and causes uncontrolled cell proliferation. Therefore, CDKs can lead to cancer occurrence [33]. CDK6 and CDK4, which are highly homologous enzymes, are regarded as classic cell cycle kinases that promote cell proliferation by forming complexes with D-type cyclins at the early G1 phase of the cell cycle [34]. CDK6 has been linked to the incidence of several cancers, according to recent studies. Hematologic malignancies such as acute myeloid leukemia (AML) and T-cell lymphoblastic lymphoma can be caused by CDK6 dysfunction [35, 36]. CDK6 deficiency has also been linked to breast cancer and melanoma [37, 38].

The function of CDK6 has been reported in PCa. A study found that baicalin could decrease PCa cell proliferation and that this effect would reverse when the CDK6 expression was upregulated, suggesting that CDK6 promotes proliferation [39]. Another study also found that CDK6 inhibitors (G1T28 and G1T38) had a protective role against CRPC [40]. In addition, CDK6 reportedly may have a function in the occurrence of EnzR CRPC. In this study, they showed that palbociclib, a CDK6 inhibitor, could promote EnzR LNCaP cell death [41]. Although these findings suggest that CDK6 can cause the occurrence and development of PCa, there is no direct evidence that suggests the expression of CDK6 in PCa and EnzR CRPC. In our study, we found that CDK6 expression was indeed higher in tumor tissues than in normal tissues. At the same time, its expression is higher in EnzR LNCaP and C4-2 than in LNCaP and C4-2 cell lines. Furthermore, we found that three CDK6 inhibitors could decrease CDK6 expression and cell proliferation. Our results directly illustrate that abnormal overexpression of CDK6 may lead to the occurrence of EnzR CRPC. Suppressing CDK6 expression can delay PCa progress. In addition, the result reflected that CDK6 inhibitors could be potential drugs to treat EnzR CRPC in the future.

However, our study has some limitations. First, the DEGs and hub genes identified in EnzR CRPC were from EnzR LNCaP. Despite testing the expression of the hub genes in TCGA and CPGEA databases, the result may be different in samples collected from patients with EnzR CRPC. In addition, because the LNCaP cell line does not mirror the CRPC cell line, the data we obtained may be biased. Furthermore, the hub genes included in the study were those that exhibited a link with other genes, indicating that the screening method was insufficient. Second, *CDK6*, *GRIP2*, *PREX2*, *ITGA1,* and *LAMB1* were differentially expressed in cells and samples in the database. We did not investigate their function since their expression differed between cell lines and clinical samples. They might potentially have a role in the development of PCa and EnzR CRPC. Third, we only verified the expression of the hub genes in one database: GEPIA. This may lead to the results being inconclusive. Fourth, the CDK6 inhibitor molecules also have other functions that may prevent tumor occurrence. Their ability to inhibit EnzR cell growth might be due to a variety of factors. As a result, more research is required to confirm our findings.

## Conclusion

We identified 10 hub genes among 45 DEGs from EnzR LNCaP cells. *CDK6*, a hub gene, plays an important role in the occurrence of PCa and EnzR CRPC. Three small molecules, namely apigenin, chrysin, and fisetin, can decrease CDK6 expression and suppress EnzR PCa cell proliferation. Therefore, they can be explored as potential drugs in treating EnzR CRPC in the future.

## Supplementary Information

**Additional file 1: Table S1.** The characteristic of Forty-five DEGs from three GSE datasets.

**Additional file 2: Figure S1.** The volcano map reflected the DEGs between LNCaP cells and EnzR LNCaP cells from different datasets. **A** GSE44905 **B** GSE78201 **C** GSE150807.

**Additional file 3: Figure S2.** The expression of ten hub genes in Chinese PCa patients (data from CPGEA database)

**Additional file 4: Figure S3.** The expression of CDK6 in PCa patients with different TNM tumor stages (data from TCGA database). **A** The expression of CDK6 in different Tumor (T) stage rely on TNM classification of malignant tumors. **B** The expression of CDK6 in different Node (N) stage rely on TNM classification of malignant tumors. **C** CDK6 expression in different T2 tumor stage patients. **D** CDK6 expression in different T3 tumor stage patients.

**Additional file 5: Figure S4.** The association of PCa and methylation level of CDK6 **A** The CDK6 methylation level in PCa patients **B** Correlation of CDK6 methylation and mRNA expression in PCa patients **C**-**D** The role of methylation CDK6 in PCa patients’ OS and DFS. (data from TCGA database).

**Additional file 6: Figure S5.** The expression of CDK6 in EnzR C4-2 (C4-2R) and 22Rv1 PCa cell lines. **A**-**B** The expression of CDK6 between C4-2 and C4-2R cells from GSE151083 and GSE136130. **C**-**D** The mRNA and protein level of CDK6 in 22Rv1 cells treated or not treated by Enz. **represents *P*<0.01, ***represents *P*<0.001. The data were shown in Mean±SD. The qRT-PCR and western blot using β-tubulin as inner control.

**Additional file 7: Figure S6.** The association of CDK6 and immune cells in PCa (data from TIMER).

**Additional file 8: Figure S7.**The correlation between nine hub genes’ expression and the prognosis of PCa in OS status got from GEPIA online tool. **A** *GRIP2* **B** *EPHB2* **C** *CDK6* **D** *PAX6* **E** *BMP7* **F** *ITGA1* **G** *LAMB1* **H** *IGFBP5* **I** *LY6K.*

**Additional file 9: Figure S8.** The ten CDK6 inhibitors got from CLUE COMMAND.

**Additional file 10: Figure S9.** CDK6 inhibitors can decrease the expression of CDK6 and cell proliferation in 22Rv1 cells. **A**-**B** The mRNA and protein level of CDK6 after 22Rv1 cells treated by CDK6 inhibitors (Apigenin, Chrysin, and Fisten). **C** 22Rv1 cell proliferation ability was detected after added CDK6 inhibitors. *represents* P*< 0.05, **represents *P*<0.01. The data were shown by Mean±SD. The qRT-PCR and western blot using β-tubulin as inner control.

## Data Availability

The datasets supporting the conclusions of this article are available in the TCGA-PRAD (https://portal.gdc.cancer.gov/) CPGEA (http://www.cpgea.com/) and CLUE COMMAND (https://clue.io/command) databases. Other data can be collected from the corresponding author.

## Abbreviations

PCa: Prostate cancer
ADT: Androgen deprivation therapy
DEGs: Differentially expressed genes
AR: Androgen receptor
CRPC: Castration-resistant prostate cancer
GEO: Gene Expression Omnibus
GO: Gene oncology
KEGG: Kyoto Encyclopedia of Genes and Genomes
PPI: Protein–protein interaction
TCGA: The Cancer Genome Atlas
GEPIA: Gene expression profiling interactive analysis platform
CPGEA: Chinese Prostate Cancer Genome and Epigenome Atlas
OS: Overall survival
DFS: Disease-free survival
TNM: Tumor-Node-Metastasis
CDK6: Cyclin-dependent kinases6
