# Supplementary material for: CDK6 is upregulated and may be a potential therapeutic target in enzalutamide-resistant castration-resistant prostate cancer
Source: Eur J Med Res. 2022 Jul 2;27:105. doi: 10.1186/s40001-022-00730-y (PMC9250190; doi:10.1186/s40001-022-00730-y)
Supplement: Supplementary file 1 — Additional file 1: Table S1. The characteristic of Forty-five DEGs from three GSE datasets. [file 40001_2022_730_MOESM1_ESM.docx]

Table S1: The characteristic of Forty-five DEGs from three GSE datasets

| Gene name | Expression trend | P-value GSE44905 | P-value GSE78201 | P-value GSE150807 |
| --- | --- | --- | --- | --- |
| CSRNP3 | Up regulated | 2.04E-05 | 4.68E-02 | 5.00E-05 |
| DTX3 | Up regulated | 3.37E-03 | 4.68E-03 | 5.00E-05 |
| CHAF1A | Up regulated | 1.65E-02 | 1.32E-02 | 4.80E-05 |
| FLT3LG | Up regulated | 4.95E-02 | 1.90E-02 | 2.08E-02 |
| LAMB1 | Up regulated | 1.95E-02 | 2.13E-02 | 5.03E-05 |
| ATAD3B | Up regulated | 2.03E-02 | 9.98E-04 | 4.72E-04 |
| BICC1 | Up regulated | 2.04E-02 | 5.68E-03 | 1.09E-02 |
| MDGA2 | Up regulated | 2.13E-02 | 5.32E-03 | 4.82E-02 |
| DNAH11 | Up regulated | 2.16E-02 | 1.17E-03 | 5.04E-05 |
| KIF25 | Up regulated | 1.14E-02 | 3.57E-02 | 2.28E-02 |
| GRIP2 | Up regulated | 2.31E-02 | 2.58E-02 | 5.41E-03 |
| CORIN | Up regulated | 2.45E-02 | 2.21E-02 | 2.46E-03 |
| FSTL4 | Up regulated | 2.67E-02 | 1.93E-02 | 5.12E-03 |
| FZD2 | Up regulated | 2.71E-02 | 1.12E-03 | 2.61E-02 |
| PDSS1 | Up regulated | 2.77E-02 | 1.24E-02 | 2.43E-02 |
| ITGA1 | Up regulated | 2.86E-02 | 4.29E-03 | 6.57E-03 |
| NAV2 | Up regulated | 2.86E-02 | 1.23E-04 | 1.51E-03 |
| ITGB1BP1 | Up regulated | 2.91E-02 | 3.78E-03 | 5.14E-05 |
| HOXA3 | Up regulated | 2.99E-02 | 1.04E-03 | 2.11E-04 |
| PAX6 | Up regulated | 3.13E-02 | 7.81E-03 | 5.21E-05 |
| LY6K | Up regulated | 3.28E-02 | 7.73E-03 | 1.57E-02 |
| CENPJ | Up regulated | 3.27E-02 | 2.16E-02 | 4.97E-04 |
| ACADL | Up regulated | 3.42E-02 | 2.25E-03 | 1.47E-02 |
| DCHS1 | Up regulated | 5.17E-05 | 3.53E-02 | 1.01E-02 |
| RBMS1 | Up regulated | 3.56E-02 | 3.81E-02 | 4.18E-03 |
| IGFBP5 | Up regulated | 5.14E-05 | 3.82E-02 | 3.92E-03 |
| FRMD4A | Up regulated | 3.99E-02 | 4.18E-02 | 5.57E-04 |
| UNC13A | Up regulated | 4.11E-02 | 5.36E-03 | 3.07E-02 |
| GART | Up regulated | 4.18E-02 | 5.14E-03 | 7.61E-03 |
| CDK6 | Up regulated | 5.07E-05 | 1.09E-05 | 4.23E-02 |
| EPHB2 | Up regulated | 5.15E-05 | 4.29E-02 | 3.79E-02 |
| RPP25 | Up regulated | 5.32E-03 | 1.55E-02 | 4.31E-02 |
| MOXD1 | Up regulated | 5.12E-04 | 1.54E-02 | 4.32E-02 |
| SEMA6D | Up regulated | 5.52E-03 | 8.98E-03 | 4.49E-02 |
| LINC01126 | Up regulated | 4.55E-02 | 5.23E-06 | 1.51E-04 |
| ZSCAN30 | Up regulated | 4.58E-02 | 8.34E-03 | 1.11E-04 |
| TNFRSF9 | Up regulated | 2.11E-04 | 1.37E-02 | 4.59E-02 |
| BMP7 | Up regulated | 4.63E-02 | 1.31E-03 | 5.02E-05 |
| DRP2 | Up regulated | 4.72E-02 | 1.74E-02 | 4.32E-04 |
| TMEM169 | Up regulated | 4.78E-02 | 8.22E-03 | 7.41E-03 |
| SLC2A3 | Up regulated | 4.82E-02 | 8.33E-03 | 6.32E-05 |
| TGFB1 | Up regulated | 4.93E-02 | 4.37E-05 | 6.11E-03 |
| PREX2 | Up regulated | 4.95E-02 | 4.88E-03 | 6.65E-03 |
| CEL | Down regulated | 8.76E-03 | 1.14E-02 | 1.13E-02 |
| STC1 | Down regulated | 5.15E-05 | 1.12E-04 | 2.37E-02 |
