# Supplementary figures and images for: CDK6 is upregulated and may be a potential therapeutic target in enzalutamide-resistant castration-resistant prostate cancer
Source: Eur J Med Res. 2022 Jul 2;27:105. doi: 10.1186/s40001-022-00730-y (PMC9250190; doi:10.1186/s40001-022-00730-y)

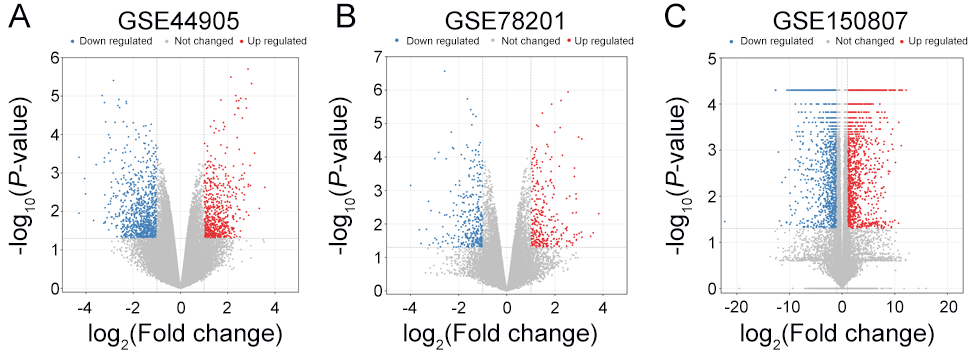

Supplement: Supplementary file 2 — Additional file 2: Figure S1. The volcano map reflected the DEGs between LNCaP cells and EnzR LNCaP cells from different datasets. A GSE44905 B GSE78201 C GSE150807. [file 40001_2022_730_MOESM2_ESM.tif]

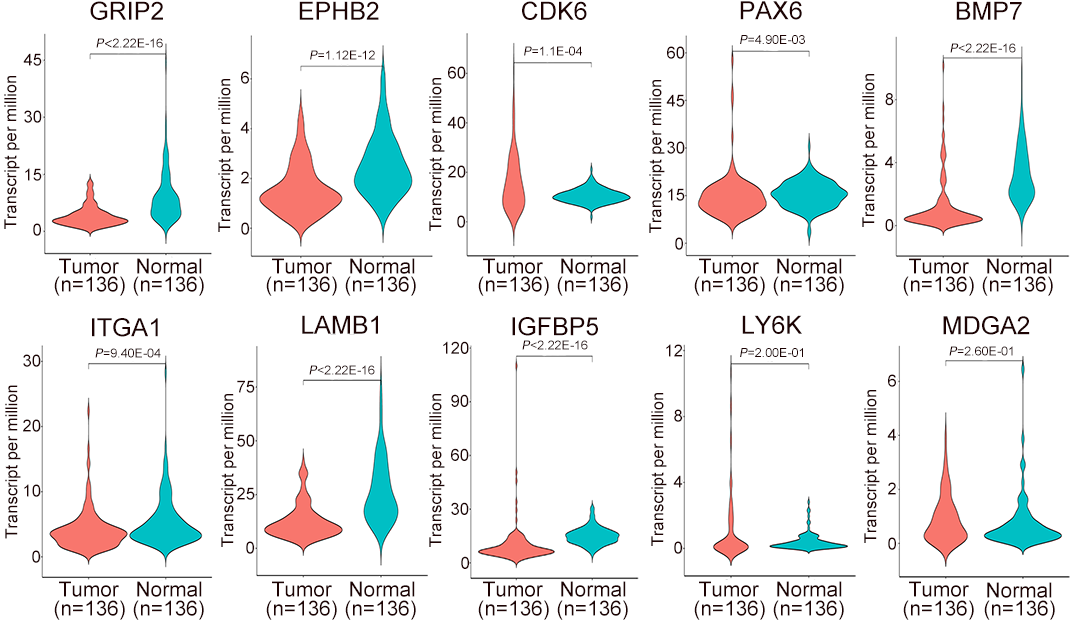

Supplement: Supplementary file 3 — Additional file 3: Figure S2. The expression of ten hub genes in Chinese PCa patients (data from CPGEA database) [file 40001_2022_730_MOESM3_ESM.tif]

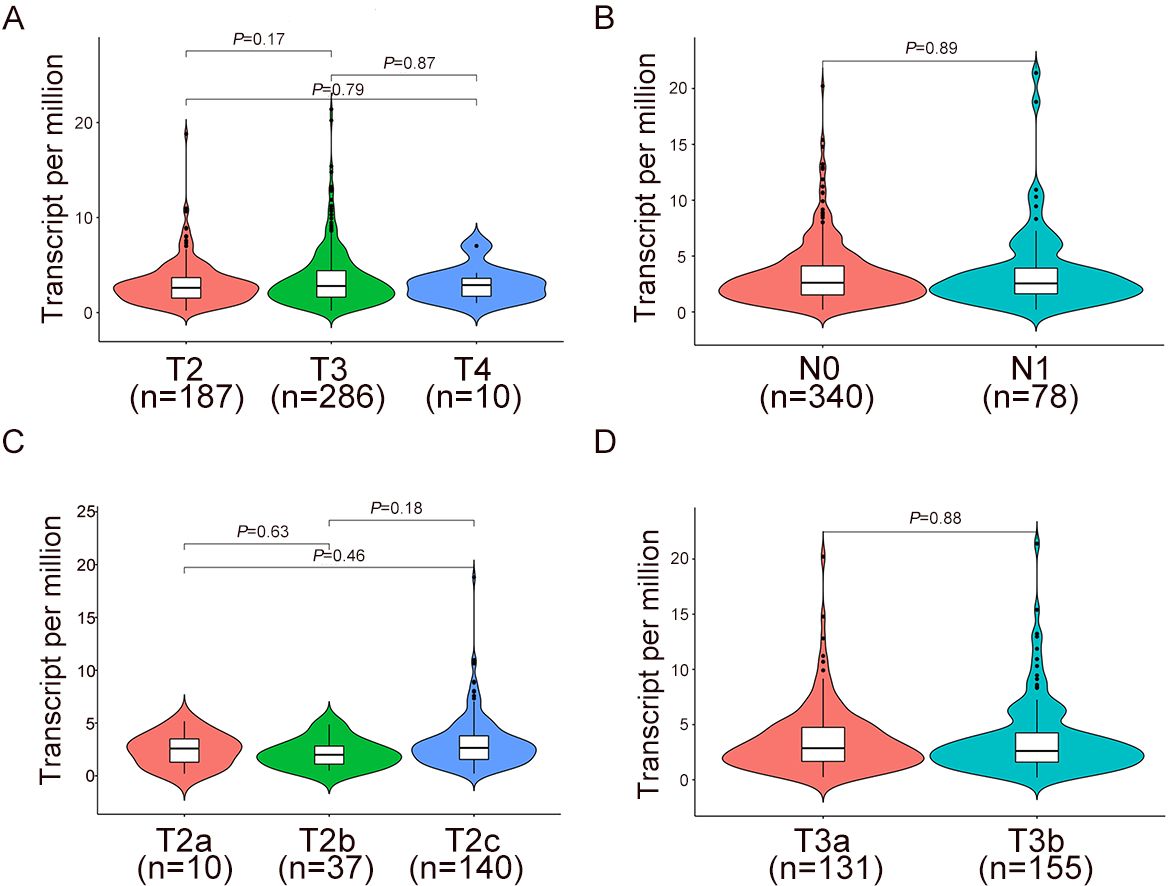

Supplement: Supplementary file 4 — Additional file 4: Figure S3. The expression of CDK6 in PCa patients with different TNM tumor stages (data from TCGA database). A The expression of CDK6 in different Tumor (T) stage rely on TNM classification of malignant tumors. B The expression of CDK6 in different Node (N) stage rely on TNM classification of malignant tumors. C CDK6 expression in different T2 tumor stage patients. D CDK6 expression in different T3 tumor stage patients. [file 40001_2022_730_MOESM4_ESM.tif]

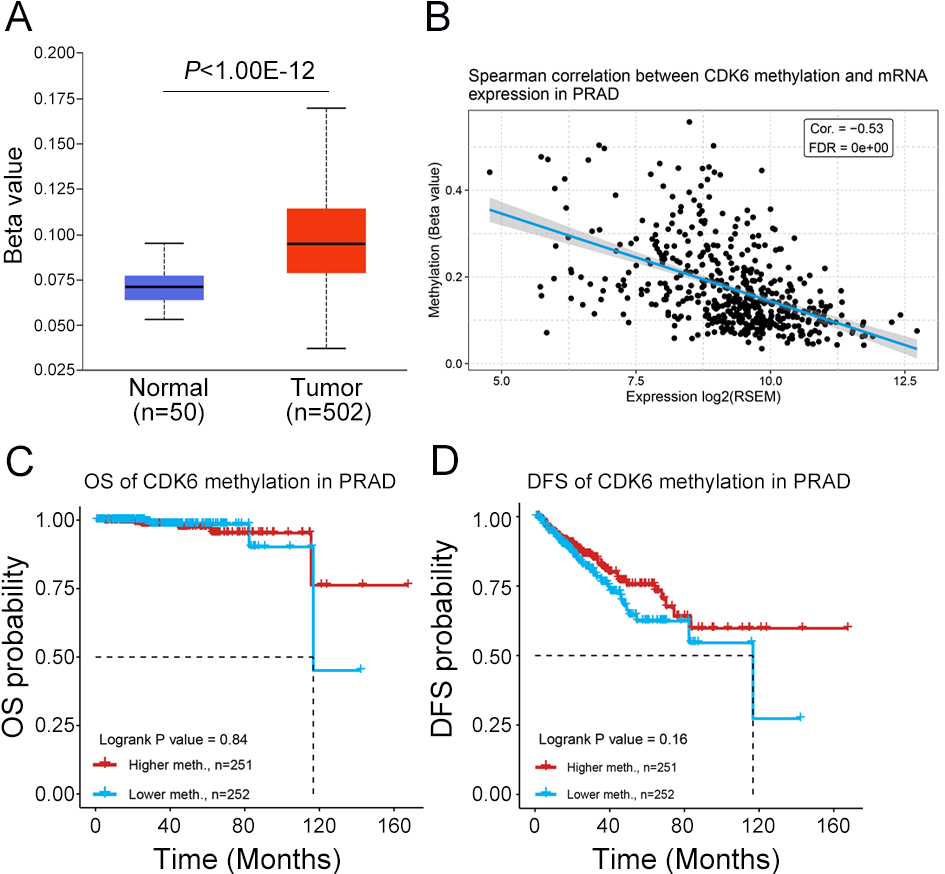

Supplement: Supplementary file 5 — Additional file 5: Figure S4. The association of PCa and methylation level of CDK6 A The CDK6 methylation level in PCa patients B Correlation of CDK6 methylation and mRNA expression in PCa patients C-D The role of methylation CDK6 in PCa patients’ OS and DFS. (data from TCGA database). [file 40001_2022_730_MOESM5_ESM.tif]

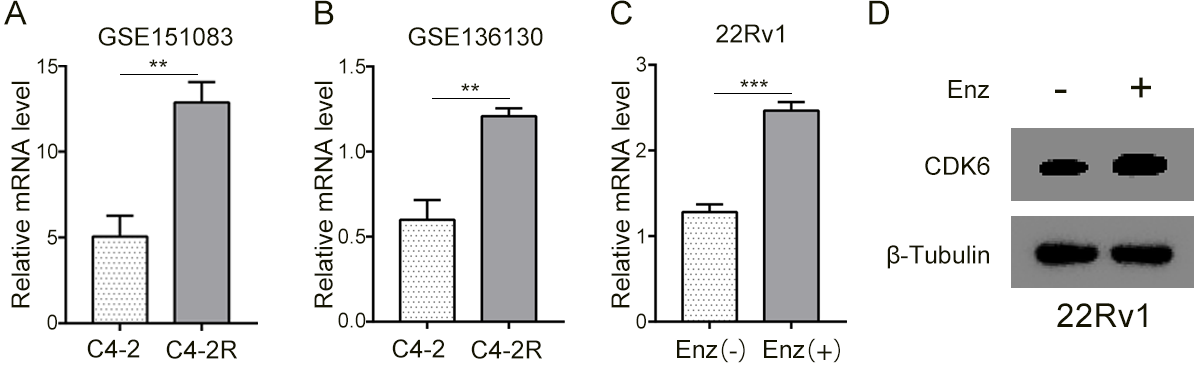

Supplement: Supplementary file 6 — Additional file 6: Figure S5. The expression of CDK6 in EnzR C4-2 (C4-2R) and 22Rv1 PCa cell lines. A-B The expression of CDK6 between C4-2 and C4-2R cells from GSE151083 and GSE136130. C-D The mRNA and protein level of CDK6 in 22Rv1 cells treated or not treated by Enz. **represents P<0.01, ***represents P<0.001. The data were shown in Mean±SD. The qRT-PCR and western blot using β-tubulin as inner control. [file 40001_2022_730_MOESM6_ESM.tif]

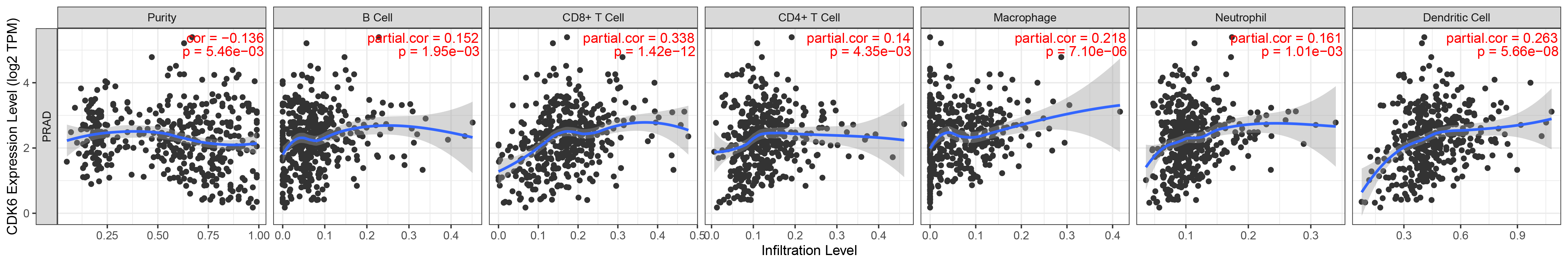

Supplement: Supplementary file 7 — Additional file 7: Figure S6. The association of CDK6 and immune cells in PCa (data from TIMER). [file 40001_2022_730_MOESM7_ESM.tif]

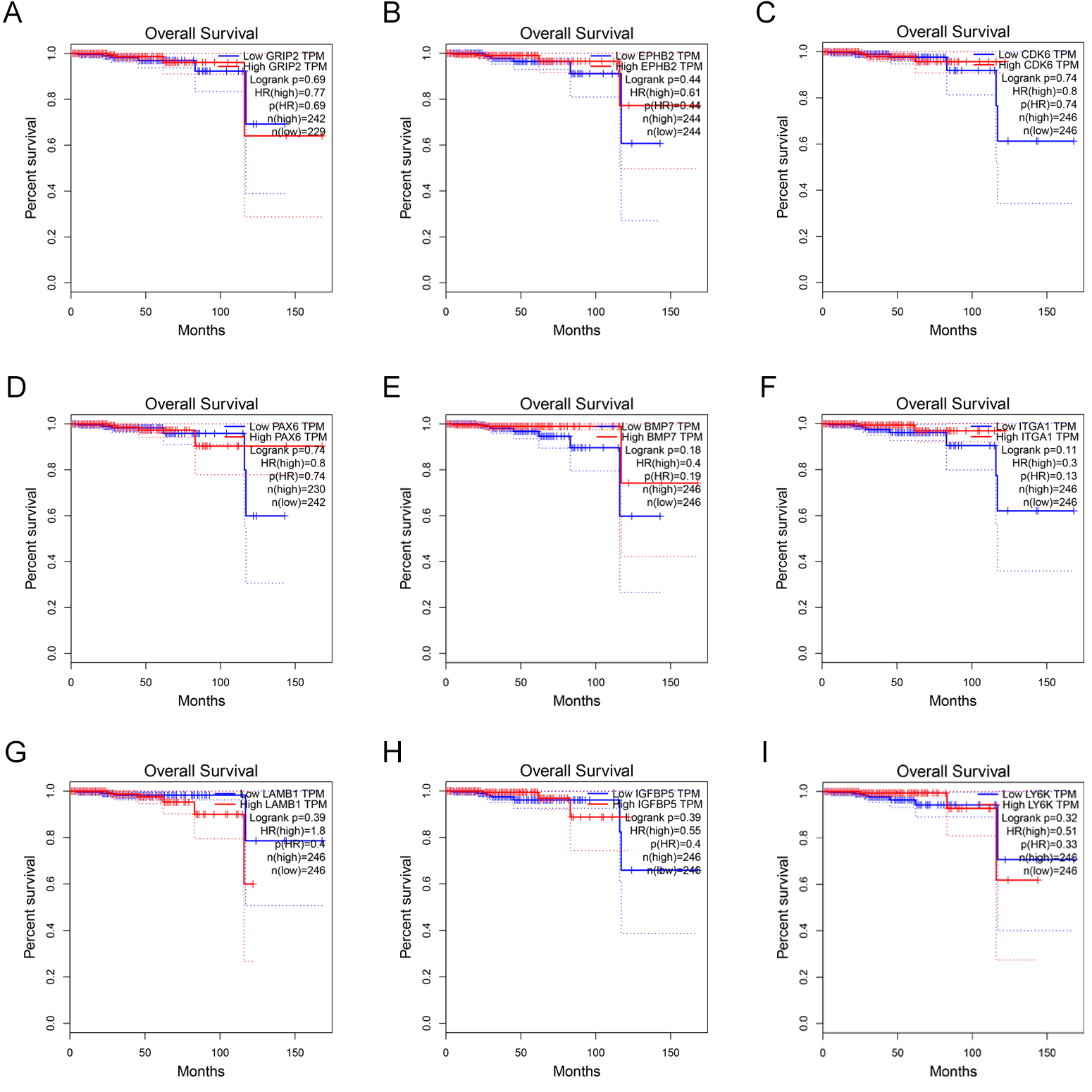

Supplement: Supplementary file 8 — Additional file 8: Figure S7.The correlation between nine hub genes’ expression and the prognosis of PCa in OS status got from GEPIA online tool. A GRIP2 B EPHB2 C CDK6 D PAX6 E BMP7 F ITGA1 G LAMB1 H IGFBP5 I LY6K. [file 40001_2022_730_MOESM8_ESM.tif]

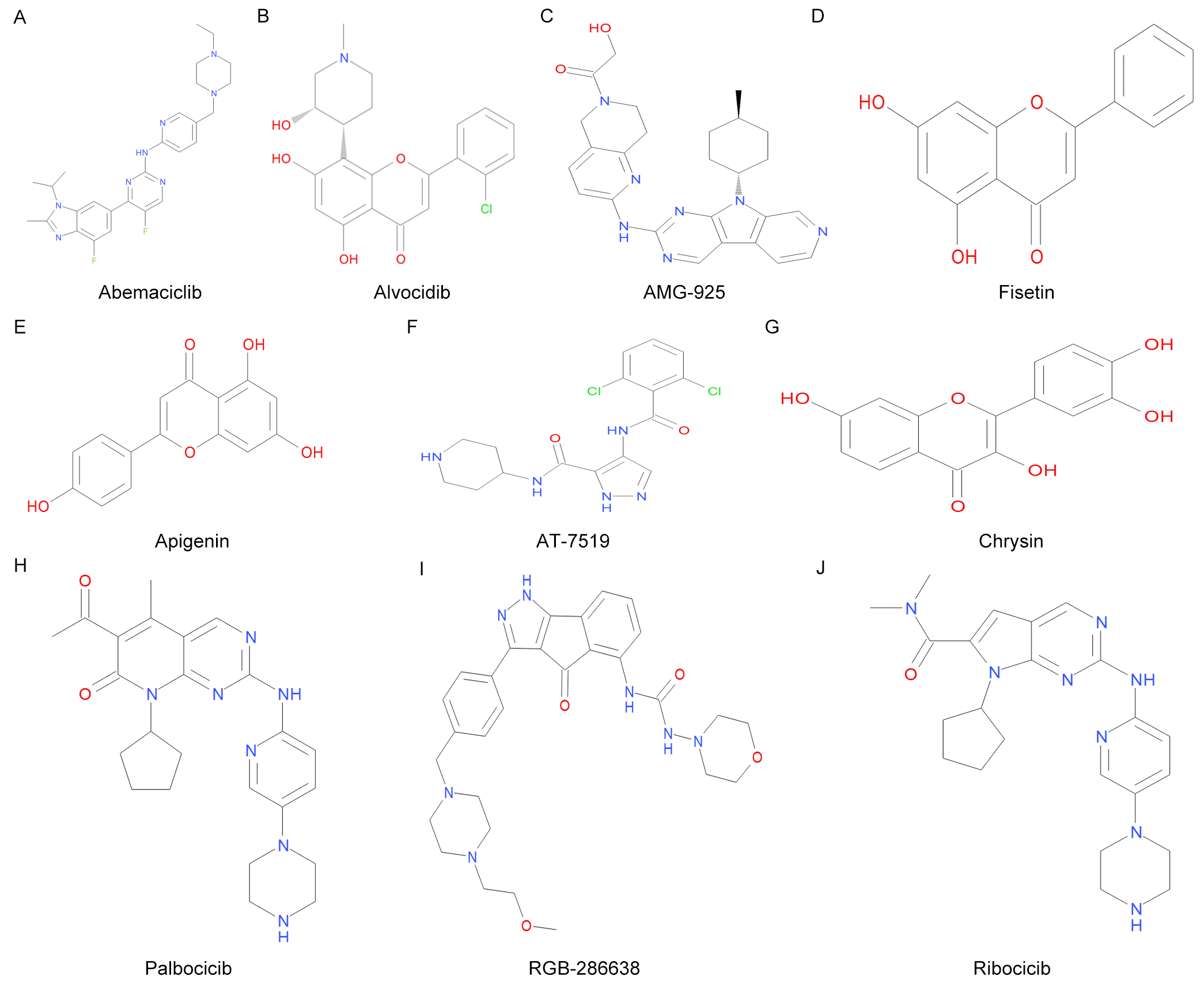

Supplement: Supplementary file 9 — Additional file 9: Figure S8. The ten CDK6 inhibitors got from CLUE COMMAND. [file 40001_2022_730_MOESM9_ESM.tif]

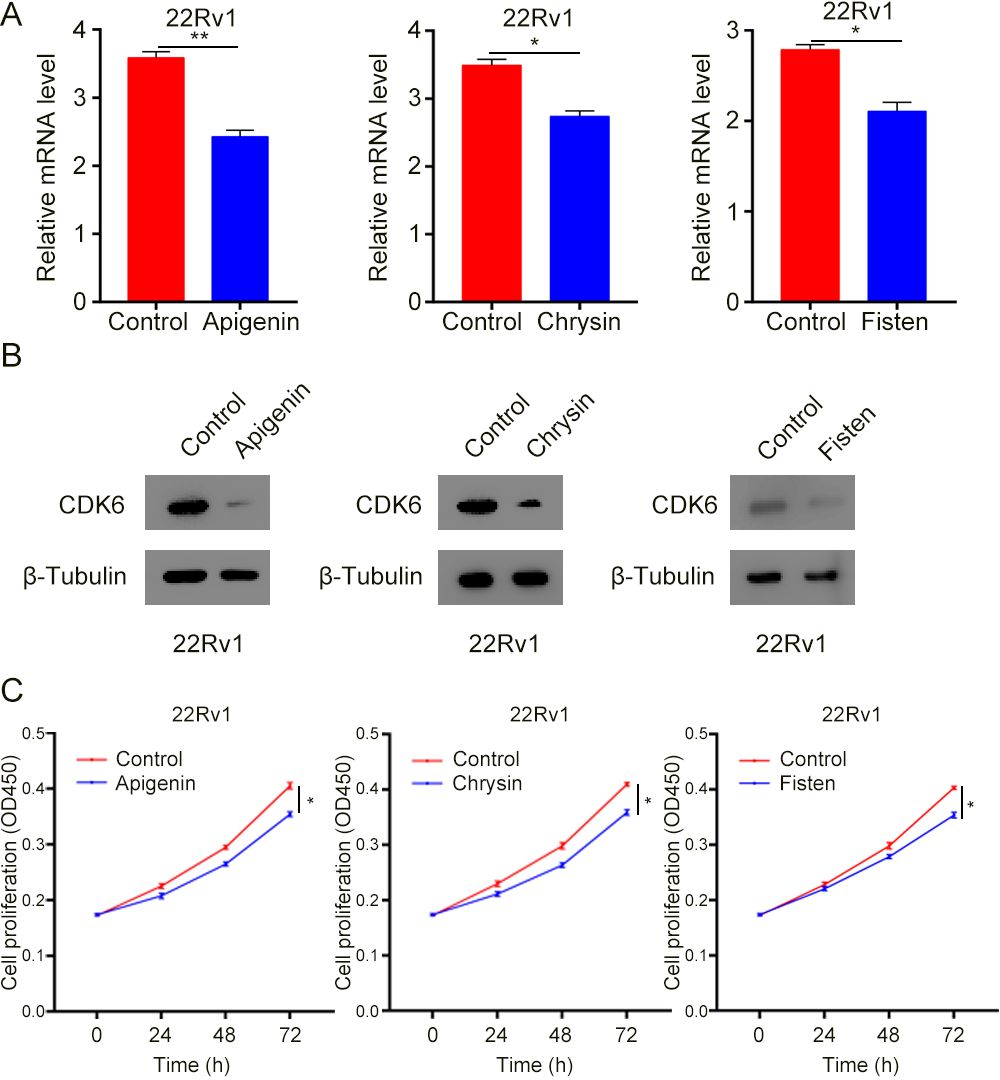

Supplement: Supplementary file 10 — Additional file 10: Figure S9. CDK6 inhibitors can decrease the expression of CDK6 and cell proliferation in 22Rv1 cells. A-B The mRNA and protein level of CDK6 after 22Rv1 cells treated by CDK6 inhibitors (Apigenin, Chrysin, and Fisten). C 22Rv1 cell proliferation ability was detected after added CDK6 inhibitors. *represents P< 0.05, **represents P<0.01. The data were shown by Mean±SD. The qRT-PCR and western blot using β-tubulin as inner control. [file 40001_2022_730_MOESM10_ESM.tif]
